# A large 15 - year database analysis on the influence of age, gender, race, obesity and income on hospitalization rates due to stone disease

**DOI:** 10.1590/S1677-5538.IBJU.2015.0743

**Published:** 2016

**Authors:** Marcos F. Mello, Giovanni Scala Marchini, Cesar Câmara, Alexandre Danilovic, Renata Levy, José Eluf-Neto, Miguel Srougi, Eduardo Mazzucchi

**Affiliations:** 1 Seção de Endourologia, Divisão de Urologia do Hospital das Clínicas da Universidade de São Paulo Faculdade de Medicina de São Paulo, Brasil; 2 Departamento de Medicina Preventiva da Universidade de São Paulo Faculdade de Medicina de São Paulo, Brasil

**Keywords:** Disease, Calculi, Ureter, Kidney Calculi, Urolithiasis

## Abstract

**Purpose:**

To assess the public hospitalization rate due to stone disease in a large developing nation for a 15-year period and its association with socio-demographic data.

**Materials and Methods:**

A retrospective database analysis of hospitalization rates in the Brazilian public health system was performed, searching for records with a diagnosis code of renal/ureteral calculi at admission between 1998–2012. Patients managed in an outpatient basis or private care were excluded. Socio-demographic data was attained and a temporal trend analysis was performed.

**Results:**

The number of stone-related hospitalizations increased from 15.7%, although the population-adjusted hospitalization rate remained constant in 0.04%. Male:female proportion among hospitalized patients was stable (49.3%:50.7% in 1998; 49.2%:50.8% in 2012), though there was a significant reduction in the prevalence of male hospitalizations (−3.8%;p=0.041). In 2012, 38% of hospitalized patients due to stone disease had 40–59 years-old. The ≥80 years-old strata showed the most significant decrease (−43.44%;p=0.022), followed by the 20–39 (−23.17%;p<0.001) and 0–19 years-old cohorts (−16.73%;p=0.012). Overall, the lowest relative hospitalization rates were found for yellow and indigenous individuals. The number of overweight/obese individuals increased significantly (+20.6%), accompanied by a +43.6% augment in the per capita income. A significant correlation was found only between income and obesity (R=0.64;p=0.017).

**Conclusions:**

The prevalence of stone disease requiring hospitalization in Brazil remains stable, with a balanced proportion between males and females. There is trend for decreased hospitalization rates of male, <40 and ≥80 years-old individuals. Obesity and income have a more pronounced correlation with each other than with stone *disease*.

## INTRODUCTION

Urinary stone disease is a common disorder, which distresses the economically active population. In the United States of America (USA), urolithiasis was reported to affect 1.116 per 100.000 18 to 64-year-old employees covered by two large insurance carriers in the year 2000 (1–4).

Kidney stones are not usually quiescent and typically cause patients considerable pain, ultimately leading to hospitalization. International epidemiological data suggest that the incidence and prevalence of stone disease are increasing (1–4). The probability of developing kidney stones varies according to numerous factors including age, gender, race, geographic location and body mass index (BMI). In addition, a change in the gender and age distribution of stone formers over time has been described, with an increasing participation of females and older individuals (5–7). This highlights the importance of better understanding current regional hospitalization rates in order to work on stone prevention and treatment strategies. Unfortunately, current population-based data on the frequency of kidney stone episodes in Latin nations are limited and studies describing recent national time trends are lacking.

The goals of the present study are to assess the rate of hospitalizations due to upper urinary tract calculi in Brazil over the past 15 years and to evaluate the influence of gender, age, race, income, obesity and geographic residence on urolithiasis trends.

## MATERIALS AND METHODS

### National Background

Brazil comprises a 8.5 billion-km2 area. According to Brazilian Institute of Geography and Statistics (Instituto Brasileiro de Geografia e Estatística-IBGE), the national population has reached more than 200 million people in 2014 (9). The Public Health System of Brazil, named Sistema Único de Saúde (SUS), supposedly provides health coverage for all citizens of the country. Recent data has shown approximately 47%-74% of residents truly uses resources from SUS and not from private care (10).

### Study Design and Data Source

A retrospective, population-based, cross-sectional time series analysis was performed using data derived from an administrative database of the Brazilian Public Health System (SUS).

Public Health System has a longitudinal hospital inpatient database (Sistema de Informação Hospitalar-SIH/SUS), which contains records from discharges for all cities and regions of the country (11). Each discharge includes up to five inpatient diagnoses per hospitalization. All diagnoses are coded using the International Classification of Disease (9th revision, ICD-9 until December 1997; 10th revision, ICD-10 since January 1998). Patient and socio-demographic characteristics included are gender, race, age, ZIP code of residence, and income.

The Consumer Expenditure Survey (Pesquisa de Orçamento Familiar-POF), the demographic census, and the national research by household sample (Pesquisa Nacional por Amostra de Domicílios-PNAD), all conducted by IBGE, aims to portray the Brazilian Population. Body mass index (BMI) data of the Brazilian population was extracted from the POF, which has been performed in two periods: 2002–2003 and 2008–2009. Income per capita data was extracted from PNAD and the demographic census, the latest one being performed in 2010 (12).

### Database Analysis

Hospital discharges between January 1^st^/1998 and December 31^st^/2012 were abstracted from the SIH/SUS. In 2012, Public Health System hospitals had around 326 thousand beds. All inpatient hospitalizations in the 15-year period for patients of any age with a primary or secondary diagnosis code of N20.x (calculus of kidney or ureter) were abstracted. Only patients with ureteral or renal stones were considered in the analysis.

The absolute number of inpatient hospitalizations due to stone disease per year was analyzed nationally and also separately for the five distinguish regions of the country, named South, Southeast, Midwest, North, and Northeast regions. Absolute numbers were also adjusted for local population to provide a prevalence perspective. Patients managed in an outpatient basis or those from private care were not considered in the analysis.

Demographics were summarized for the entire cohort of patients. The specific demographics included were age, gender, race, income and obesity. For population comparison, prevalence estimates were adjusted by direct standardization to the Census population. Age was reported divided in five strata (0–19; 20–39; 40–59; 60–79; and ≥80 years-old). Income was reported as national and regional income per capita per year. In regards to BMI, individuals were divided into 2 groups: normal if BMI<25Kg/m^2^; overweight and obese if BMI≥25Kg/m^2^).

### Statistical analysis

Statistical analysis was performed using SPSS V.19 (Chicago, IL). Qualitative variables were presented as absolute numbers and relative frequencies at each time point. Percent variation from study beginning to end was also contemplated. Quantitative variables were analyzed in terms of temporal trends: prevalence trends over the 15-year period were quantified by the estimated annual percent change (EAPC) using the least squares linear regression methodology (13). The linear regression model considered a 95% confidence interval with a regression coefficient. A Spearman's correlation test was performed to seek for associations between hospitalization rate, obesity, and income. Significance level was p<0.05.

## RESULTS

### Hospitalization Trends

**Figure 1:**
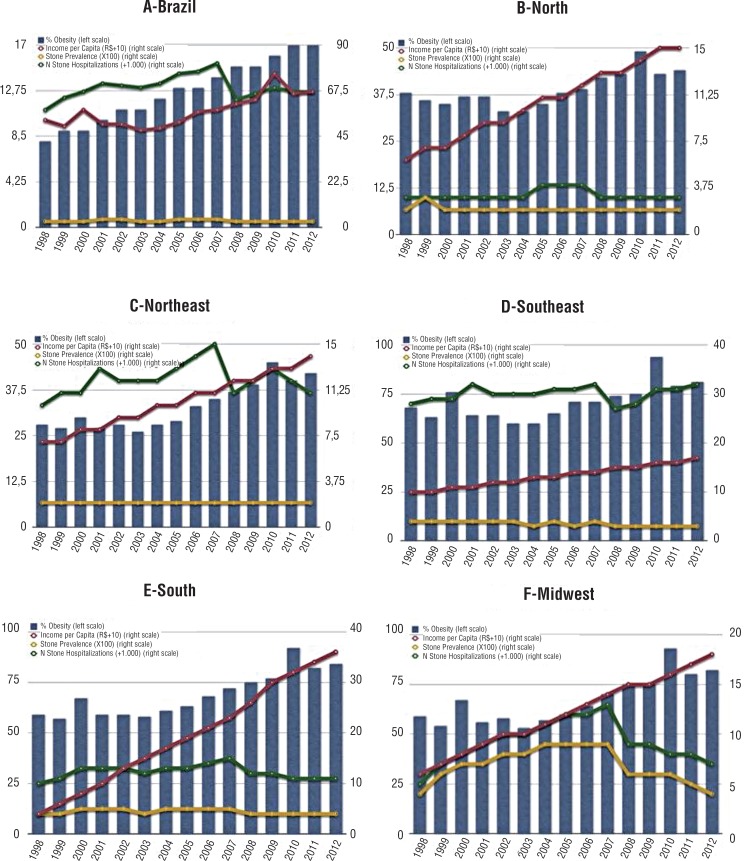
Number and Proportion of Hospitalizations in Brazil and all five regions, with % of obesity and Income Analysis.

The absolute number of stone-related hospitalizations increased from 58165 in 1998 to 67306 in 2012, accounting for a 15.7% increase in Brazil. However, the prevalence of hospitalizations adjusted for the Brazilian population remained constant in 0.04% (Table-1; Figure-1A). All regions presented a non-significant increase in the absolute number of hospitalizations (Table-1A), which was not sustained when adjusted for the local population (Table-1B).

**Table 1:** Temporal trends for absolute number (A) and prevalence (B) (adjusted to the population × 100.000 people) of stone-related hospitalizations in Brazil and all five regions between 1998 and 2012.

A		N (1998)	N (2012)	% Variation	EAPC [95% CI]	p-value
**Brazil**		58165	67306	+ 15.7%	294.7 [−454.9; 1044.3]	0.411
	Male	28675	33090	+ 15.4%	0.000% [−0.001; 0.002]	0.707
	Female	29490	34216	+ 26.2%	0.000% [−0.002; 0.001]	0.707
**Regions**						
	North	3281	3715	+ 13.2%	18.3 [−27.8; 64.4]	0.407
	Northeast	10454	11991	+ 14.7%	109.1 [−51.4; 269.7]	0.166
	Southeast	28532	32521	+ 14.0%	95.7 [−104.9; 296.3]	0.321
	South	10780	11942	+ 10.8%	−13.1 [−178.4; 152.3]	0.867
	Midwest	5118	7137	+ 39.4%	84.6 [−216.1; 385.3]	0.554
B		(1998)	(2012)	% Variation	EAPC [95% CI]	p-value
**Brazil**		35.9	34.7	−3.5%	−0.32 [−0.67; 0.04]	0.078
	Male	35.9	34.8	−3.0%	−0.0003% [−0.0006; 0.000]	**0.041**
	Female	35.9	34.6	−3.8%	−0.0003% [−0.0008; 0.0001]	0.123
**Regions**						
	North	27.6	22.7	−17.80%	−0.44 [−0.73; −0.16]	**0.005**
	Northeast	22.8	22.2	−2.52%	−0.07 [−0.37; 0.23]	0.628
	Southeast	41.3	39.8	−3.63%	−0.35 [−0.60; −0.11]	**0.009**
	South	44.6	43.1	−3.51%	−0.52 [−1.05; 0.02]	0.057
	Midwest	46.5	49.5	+ 6.30%	−0.54 [−2.81; 1.74]	0.618

The Midwest region had the highest hospitalization prevalence (0.05% in 1998 and 2012) and the highest progression rate, although non-significant (+6.2%; p=0.61; Figure-1F). The Northeast region showed the lowest hospitalization rate (0.02% in 1998 and 2012), which remained stable (−2.5%; p=0.62; Figure-1C). The only two regions with a significant change in time trends for hospitalization rates were the North (−17.8%; p=0.005) and the Southeast region (−3.63%; p=0.009), both with significant decreases (Table-1B).

### Gender Analysis

**Figure 2:**
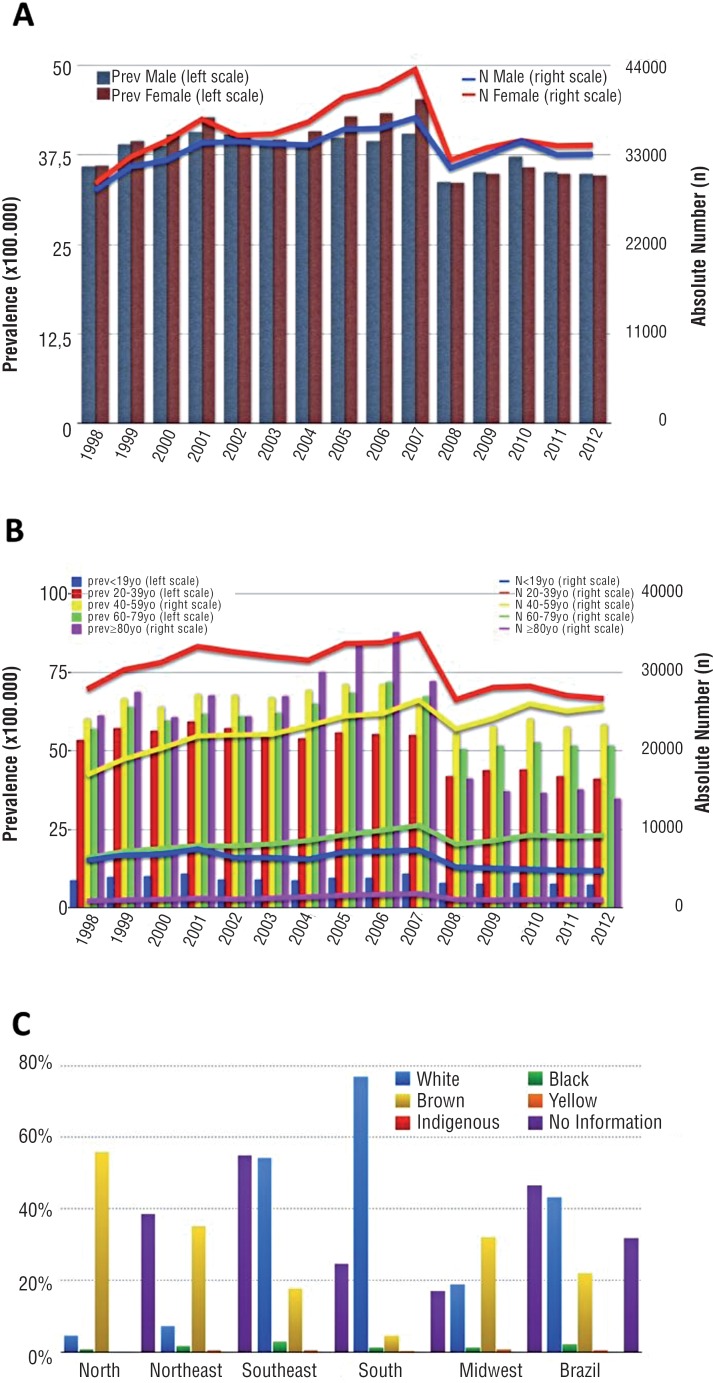
Gender (A) and Age (B) distribution among patients hospitalized due to stone disease in Brazil from 1998 to 2012; Ethnicity (c) distribution of hospitalizations for different Brazilian regions in 2012.

Male:female proportion among hospitalized patients was relatively stable in Brazil, with 49.3%:50.7% in 1998 and 49.2%:50.8% in 2012 (Figure-2A). The increase in the absolute number of hospitalizations for men (+15.4%) and women (26.2%) showed no significant estimated annual percent change (p non-significant) (Table-1A). However, there was a significant reduction in the prevalence of male hospitalizations in the analyzed period (−3.8%; p=0.041) (Table-1B).

### Age Temporal Changes

In 2012, the prevalence of hospitalizations due to stone disease in the Brazilian population stratified by population age was 7.1% from 0–19 years; 39.6% 20–39 years; 38.0% 40–59 years; 13.7% 60–79 years; and 1.5% ≥80 years (Figure-2B).

In absolute numbers, both 0–19 and 20–39 years-old strata presented a significant decrease with a negative EAPC (p<0.001), while 40–79 years-old individuals had a significant increase (p<0.001) (Table-2A). In discordance, all age strata showed a decrease in the prevalence of stone-related hospitalization rates between 1998 and 2012. However, temporal trend analysis revealed a stable situation for the 40–59 (−3.06%; p=0.06) and 60–79 years-old groups (−9.26%; p=0.087). The ≥80 years-old strata showed the most significant decrease (−43.44%; p=0.022), followed by the 20–39 years-old (−23.17%; p<0.001) and 0–19 years-old cohorts (−16.73%; p=0.012) (Table-2B).

**Table 2:** Temporal trends for absolute number (A) and prevalence (B) (adjusted to the population × 100.000 people) of stone-related hospitalizations in Brazil between 1998 and 2012 according to age strata.

A		(1998)	(2012)	% Variation	EAPC [95% CI]	p-value
**Brazil**						
	0 to 19 years	6089	4757	−21.87%	−0,26% [−0,32;0,11]	<0.001
	20 to 39 years	27856	26671	−4.25%	−0,60% [−0,65;−0,55]	<0.001
	40 to 59 years	16933	25607	+ 51.2%	0,65% [0,53;0,76]	<0.001
	60 to 79 years	6349	9240	+ 45.5%	0,21% [0,18;0,24]	<0.001
	≥ 80 years	938	1031	+ 9.91%	0,01% [−0,34;0,35]	0.976
B		(1998)	(2012)	% Variation	EAPC [95% CI]	p-value
**Brazil**						
	0 to 19 years	8.9	7.4	−16.73%	−0.16 [−0.28;−0.04]	**0.012**
	20 to 39 years	53.3	41	−23.17%	−0.12 [−1.75;−0.70]	**<0.001**
	40 to 59 years	60.1	58.2	−3.06%	−0.59 [−1.20;0.03]	0.060
	60 to 79 years	56.8	51.6	−9.26%	−0.71 [−1.54;0.12]	0.087
	≥ 80 years	61.3	34.6	−43.44%	−2.33 [−4.27;−0.39]	**0.022**

### Ethnicity Evaluation

There were no records of hospitalization by race until 2009 and no time-trend analysis could be performed. The distribution by race in 2012 was 43.4% white; 2.2% black; 22.1% brown; 0.5% yellow; 0.03% indigenous and 31.8% with no information (Figure-2C).

In 2012, the distribution by race showed two patterns: in the North, Northeast and Midwest regions, brown citizens were the most common among those hospitalized due to stone disease (55.8%, 35.2%, 32.1%, respectively). In the Southeast and South regions, there was a predominance of Caucasians (54.1% and 76.9%, respectively). There were high percentages of missing records (no information) in all regions. Overall, the lowest relative hospitalization rates were found for yellow and indigenous individuals (Figure-2C).

### Obesity, Income and Hospitalization Correlation

The number of overweight and obese individuals increased significantly (+20.6%) (Table-3A) and was accompanied by a +43.61% augment in the per capita income (Table-3B). The correlation of hospitalization rate with income (R=−0.36; p=0.22) and obesity rate (R=−0.42; p=0.11) was not significant. Conversely, a positive significant correlation was found between income and obesity (R=0.64; p=0.017) (Figure-1). Table-3 shows overweight prevalence and income per capita for Brazil and all five regions.

**Table 3:** Temporal variation of overweight prevalence (A) and income per capita (B) in Brazil.

A				
People overweight		% (2003)	% (2012)	% Variation
Brazil		40.2 %	48.5%	+ 20.6%
**Regions**				
North		35.0%	46.5%	+ 32.8%
	Northeast	35.5%	43.5%	+ 22.5%
	Southeast	42.2%	49.0%	+ 16.1%
	South	44.6%	52.3%	+ 17.3%
	Midwest	39.9%	47.5%	+ 19.0%
B				
Income per capita		US$ (1998)	US$ (2010)	% Variation
Brazil		164.72	237.00	+ 43.6%
**Regions**				
	North	119.29	152.67	+ 27.8%
	Northeast	89.22	141.31	+ 58.4%
	Southeast	210.73	291.05	+ 38.2%
	South	184.16	283.82	+ 54.2%
	Midwest	182.54	288.41	+ 58.2%

The North region showed the highest increase in the proportion of overweight people (+32.8%; 35.0% in 2003 and 46.5% in 2009) and the lowest increase on income per capita (+27.8%; US$199.29 in 1998 and US$152.67 in 2010) (Table-3). Surprisingly, there was a decrease in the incidence of hospitalization by stone disease (−17.8%; p=0.005) (Table-1B).

In Northeast region, there was an increase in relative number of overweight people (+22.5%; 35.5% in 2003 and 43.5% in 2009) and also in the income per capita (+58.4%; US$89.22 in 1998 and US$141.31 in 2010) (Table-3). Hospitalization remained stable (−2.52%; p=0.628) (Table-1B).

The Southeast region showed stability of hospitalization rates (−3.63%; p=0.009) (Table-1B), with an increase in the number of overweight individuals (+16.1%; 42.2% in 2003 and 49.0% in 2009) and in the income per capita (+38.2%; US$210.73 in 1998 and US$291.05 in 2010) (Table-3).

In the South region, there was a relative increase in total number of overweight people (+17.3%; 44.6% in 2003 and 52.3% in 2009) and in the income per capita (+54.2%; US$184.16 in 1998 and US$283.82 in 2010; Table-3), with stable rates oh hospitalization (−3.51%; p=0.057) (Table-1B).

In the Midwest region, there was a relative increase in the number of overweight citizens (+19.0%; 39.9% in 2003 and 47.5% in 2009) and in the income per capita (+58.2%; US$182.54 in 1998 and US$288.41 in 2010; Table-3). In parallel, hospitalization increased (+6.3%; p=0.618) (Table-1B).

## DISCUSSION

The importance of prevention is highlighted in an era of stone disease prevalence growth worldwide. Factors that may play a role in the increasing risk of kidney stone disease include sunlight and heat, dietary consumption of animal protein, salt and water, and certain clinical conditions like obesity. After mapping the present scenario, better policies and actions might be commenced to improve prevention. However, a standardized analysis of hospitalizations due to stone disease has never been made in Brazil. Our study tries to somehow depict the national scenario.

A number of studies have shown that the lifetime prevalence of kidney stones among adults increased significantly in the past years. The National Health and Nutrition Examination Survey showed a significant 37% increase in the prevalence of stone disease in the American population between 1976–1980 and 1988–1994 (1). Benjamin et al. used the Hospital Episode Statistics (HES) to evaluate national trends in the UK between 2000 and 2010 and found similar trends, the number of upper urinary tract stone hospital episodes increased by 63% (4). In the other hand, Ghani et al. studied the US Nationwide Inpatient Sample in the period of 1999–2009 and found that the total number of hospitalizations for upper urinary tract calculi was found relatively stable (181 592 in 1999 vs. 190 040 in 2009, EAPC 0.07%, P=0.83, 95% CI: −0.53–0.68%) (6). Importantly, their research does not account for patients treated in an outpatient basis, a common practice in the USA.

In the period studied (1998–2012), the absolute number of inpatient stone-related episodes increased 15.7% in Brazil (14). However, the prevalence of hospitalizations remained almost stable. This is in disagreement with the world literature (1–4). Possible explanations could rely in the fact that in an economic growth scenario, more people are gaining access to private care treatment and not using the public health system. In addiction, a combined analysis of stone disease treatment including inpatient and ambulatory cases could show different results. Unfortunately, we do not have in Brazil a comprehensive private and outpatient database to address this matter.

The prevalence of stone disease increases with age and is relatively uncommon before 20 years-old. However, the incidence increases rapidly and peaks from ages 40 to 60 years and then decreases from ages 65 years and thereafter (2). We found that all age strata presented a significant decrease in hospitalization prevalence, with the exception of 40–59 and 60–79 years-old populations. In other words, while young individuals are less hospitalized, older ones have stable rates of hospitalization. This could be a result of the direct or indirect influence of obesity, metabolic syndrome and diabetes mellitus.

Physiologically, obesity has been linked to increased renal excretion of calcium and uric acid, as well as increased urine acidity, all of which increase the risk of stone formation. Presuming obesity as a marker for the metabolic syndrome, which is linked epidemiologically and physiologically to risk of kidney stones, we investigated potential associations between obesity and a history of kidney stones. We found a significant increase in overweight and obese individuals in the studied period (+20.6%). A previous study preformed by Taylor et al. determined that body size is independently associated with the development of kidney stones (15). The authors showed a relative risk of kidney stones in obese men to be 1.33 vs that in normal weight men, whereas in obese women the risk was up to 2.09 times higher than in normal weight women. In their study, body mass, weight gain in adulthood, and waist circumference were associated with an increased risk of incident stones. Women were at a higher risk for stone disease than men for all of these risk factors. In our investigation, a significant correlation was only found between stone episodes and income per capita. No association between obesity and stone disease prevalence could be found. Again, the analysis of private care could alter these findings.

Historically, men have been more likely to develop urinary calculi with an incidence and prevalence of up to 2–4 times greater than that for women (16, 17). In the present study, women and men had similar proportion in regards to hospitalization due to upper urinary tract calculi. In accordance to literature data, although we found a non-significant increase in the absolute number of male (+15.4%) and female (+26.2%) hospitalizations, there was a significant reduction in the prevalence of male hospitalization rates (−3.8%; p=0.041). Scales et al. showed that the ratio of males to females treated as inpatients for stone disease changed from 1.7:1 in 1997 to 1.3:1 in 2002 (5). Based on self-reports of stone disease, Stamatelou et al. showed a larger increase in women rates than in men between 1976 and 1994 (1).

Mean average temperature is believed to be a major contributor to variation in geographic risk for stone disease, since higher average temperatures and greater sun exposure could result in oversaturation of stone-forming salts in the urine. In our study, however, the sunnier and heater regions (North and Northeast) showed the lowest hospitalization rates. Those two regions are also the poorer regions of the country and this finding could reflect the difficulty in access to health coverage. The most developed regions of Brazil (South, Southeast) showed the highest hospitalization rates due to stone disease. In addition, individuals living in developed areas have a different lifestyle of people living in underdeveloped areas and they tend to feed on processed food and to be more sedentary. Thus, the explanation for the geographic variation in kidney stone risk is probably multifactorial in our country.

Potential limitations to our study must be stressed. SIH/SUS is a snapshot of a patient's admission and the study does not distinguish patients who may have had multiple admissions for the same or a new calculus episode. Patients discharged from the emergency room, those undergoing outpatient treatments or those who are managed in the supplementary system are not captured. Also, the database used does not distinguish elective from emergency hospitalizations and the accuracy of coding for procedures is unclear. Between 1998 and 2012, extra corporeal shock-wave lithotripsy had a significant relative increase (+338%) what may have lead to a decrease of number of hospitalization, since SWL can be performed in and outpatient basis (14). Another drawback was that in 2008 a new form of register for hospitalizations was implemented, leading to a potential coding bias. Lastly, the nature of the dataset does not allow the true capturing of risk factors and we can only hypothesize on causality. Importantly, this is not a phenomenon exclusive of Brazil. Our analysis included all inpatient hospitalizations for stone disease performed in SUS in the analyzed period and provided an accurate picture of local and regional stone disease tendencies.

## CONCLUSIONS

The prevalence of stone disease requiring hospitalization in Brazil remains stable, with a balanced proportion between males and females. There is trend for decreased hospitalization rates of male, <40 and ≥80 years-old individuals. Obesity and income have a more pronounced correlation with each other than with stone disease.

## References

[1] Stamatelou KK, Francis ME, Jones CA, Nyberg LM, Curhan GC (2003). Time trends in reported prevalence of kidney stones in the United States: 1976–1994. Kidney Int..

[2] Romero V, Akpinar H, Assimos DG (2010). Kidney stones: a global picture of prevalence incidence, and associated risk factors. Rev Urol..

[3] Scales CD, Smith AC, Hanley JM, Saigal CS, Urologic Diseases in America Project (2012). Prevalence of kidney stones in the United States. Eur Urol..

[4] Turney BW, Reynard JM, Noble JG, Keoghane SR (2012). Trends in urological stone disease. BJU Int..

[5] Scales CD, Curtis LH, Norris RD, Springhart WP, Sur RL, Schulman KA (2007). Changing gender prevalence of stone disease. J Urol..

[6] Ghani KR, Sammon JD, Karakiewicz PI, Sun M, Bhojani N, Sukumar S (2013). Trends in surgery for upper urinary tract calculi in the USA using the Nationwide Inpatient Sample: 1999–2009. BJU Int..

[7] Strope SA, Wolf JS, Hollenbeck BK (2010). Changes in gender distribution of urinary stone disease. Urology.

[8] Saigal CS, Joyce G, Timilsina AR, Urologic Diseases in America Project (2005). Direct and indirect costs of nephrolithiasis in an employed population: opportunity for disease management?. Kidney Int..

[9] IBGE (2014). Brazilian Institute of Geography and Statistics.

[10] Porto SM, Ugá MA, Moreira Rda S (2011). An analysis of use of the health services by financing system: Brazil 1998–2008. Cien Saude Colet..

[11] SIH/SUS (2012). Hospital Information System of Public Health System of Brazil.

[12] POF (2014). Consumer Expenditure Survey from Brazilian Institute of Geography and Statistics; 2002–2003 and 2008–2009.

[13] Anderson WF, Camargo MC, Fraumeni JF, Correa P, Rosenberg PS, Rabkin CS (2010). Age-specific trends in incidence of noncardia gastric cancer in US adults. JAMA.

[14] Marchini GS, Mello MF, Levy R, Vicentini FC, Torricelli FC, Eluf-Neto J (2015). Contemporary Trends of Inpatient Surgical Management of Stone Disease: National Analysis in an Economic Growth Scenario. J Endourol..

[15] Mattix Kramer HJ, Grodstein F, Stampfer MJ, Curhan GC (2003). Menopause and postmenopausal hormone use and risk of incident kidney stones. J Am Soc Nephrol..

[16] Heller HJ, Sakhaee K, Moe OW, Pak CY (2002). Etiological role of estrogen status in renal stone formation. J Urol..

[17] Taylor EN, Stampfer MJ, Curhan GC (2005). Obesity, weight gain, and the risk of kidney stones. JAMA.

